# GastroGPT Pioneering Specialized AI in Gastroenterology: Strengths, Pitfalls, and the Road to Clinical Integration

**DOI:** 10.1002/jgh3.70306

**Published:** 2025-11-16

**Authors:** Angad Tiwari, Hareesha Rishab Bharadwaj, Khabab Abbasher Hussien Mohamed Ahmed, Dushyant Singh Dahiya

**Affiliations:** ^1^ Department of Internal Medicine Maharani Laxmi Bai Medical College Jhansi Uttar Pradesh India; ^2^ Royal Stoke University Hospital University Hospitals of North Midlands NHS Trust Stoke‐On‐Trent Staffordshire UK; ^3^ Department of Medicine The University of Khartoum Khartoum Sudan; ^4^ Division of Gastroenterology, Hepatology & Motility The University of Kansas School of Medicine Kansas City Kansas USA

**Keywords:** artificial intelligence, gastroenterology, GastroGPT, large language models

## Abstract

GastroGPT, a transformer‐based large language model, has been developed specifically for gastroenterology. It exhibited improved ability in clinical tasks compared to a general‐purpose model such as GPT‐4, Bard and Claude. GastroGPT was developed by Cem Simsek, MD, and was presented at UEG Week 2023. The GastroGPT dataset is adapted on 1.2 million tokens, including peer‐reviewed content from leading gastroenterology journals, clinical guidelines and 10 000 synthetic GI vignettes. In 10 simulated cases of inflammatory bowel disease, cases of endoscopy, and hepatology, GastroGPT achieved a mean score of 8.1 ± 1.8 on a 10‐point Likert scale. GastroGPT achieved higher mean scores compared to comparators (*p* < 0.001) on six out of seven tasks and included tasks such as patient history acquisition, recommendation for referral, and patient education. Its reproducibility and consistency across task complexities indicate its potential in situations of resource limitation. While it remains limited by its reliance on simulated cases, some participant selection and exposure bias attributed to training data, and lack of appropriate comparisons with medical‐specific models such as OpenEvidence, there remains the need for future real‐world trials and multimodal integrations within workflows to evaluate GastroGPT's transformation potential in improving gastroenterology workflows and patient care.

## 1 Introduction

Artificial intelligence (AI) has been recognized as a disruptive technology in healthcare and has been shown to be significantly successful in several areas (e.g., medical imaging, genomic analysis), with its adoption increasing with the recent introduction of large language models (LLMs), which are applicable to a wide range of clinical applications including decision support, documentation, and patient education [1, 2]. In the field of gastroenterology, for example, average wait times for a specialist appointment exceed more than 65 days for higher income regions and other options may not exist. Therefore, AI can help to create efficiencies and eliminate access barriers [3, 4]. GastroGPT, a proof of concept large language model specifically developed for gastroenterology, was presented by Cem Simsek, MD, from Hacettepe University and debuted at UEG Week 2023 in Copenhagen with superior performance (mean score 8.1 ± 1.8) compared to general models (like GPT‐4) on clinical tasks including diagnosis and patient management [5, 6]. GastroGPT is based on a transformer‐based architecture and was tuned on a 1.2 million token dataset from peer‐reviewed journals and clinical guidelines, as well as 10 000 synthetic GI vignettes [6]. Simsek et al. demonstrate its potential to transform GI workflows as a proof of concept [6].

The model was assessed in a blinded, controlled design against general‐purpose LLMs such as GPT‐4 (LLM‐A), Bard (LLM‐B), and Claude (LLM‐C), across 10 simulated cases that included varying complexities, frequencies, and subspecialties such as IBD, endoscopy, and hepatology [6]. Performance was evaluated across seven clinical tasks, which involved assessment and summarization, additional history gathering, recommended studies, proposed management, follow‐up recommendations, referral guidance, and patient counseling. GastroGPT demonstrated a mean overall score of 8.1 ± 1.8 on a 10‐point Likert scale that was significantly superior to GPT‐4 (5.2 ± 3.0), Bard (5.7 ± 3.3), and Claude (7.0 ± 2.7) (*p* < 0.001 for all) [6]. It was superior at six of the seven tasks with lower variance (34.95 vs. 97.4–260.35), suggesting more consistency in scores across scenarios [6]. This paper evaluates GastroGPT's strengths, limitations, and implications, arguing that while specialized LLMs offer transformative potential, their clinical utility requires rigorous validation, including comparisons to emerging medical‐specific models.

## 2 Strengths of GastroGPT: A Step Toward Precision AI

GastroGPT's case‐specific fine‐tuning enables it to adeptly manage gastroenterology‐specific clinical tasks, consistently exceeding the performance of more general models. Extending the analogy of the study's topographic representation, general LLMs (landscapes) and specialist models (very narrowly defined ridges), GastroGPT is uniquely positioned to complete gastroenterology‐specific clinical tasks with great precision, and its superior performance is supported by 13 board‐certified gastroenterologists (intraclass correlation coefficient of 0.89) [6]. GastroGPT significantly outperformed general models in additional history gathering (8.43 ± 1.83 vs. 2.84–2.98; *p* < 0.001), referral guidance (8.30 ± 1.67), and patient counseling (8.50 ± 1.73) [6]. GastroGPT also showed a high level of consistency in its performance despite increases in case complexity (e.g., 7.9 ± 1.8 in high complexity cases versus divergences of 5.1–6.8 for comparators), which has implications for the potential applicability with conditions in resource‐limited circumstances [6]. In addition to its performance results, GastroGPT can readily access shareable web‐based buttons for summation, diagnostic plans and patient information sheets, which support effortless integration into clinical practice. A comparison of GastroGPT's performance details has been presented in Table 1, which highlights differences in performance compared to general LLMs. Overall, these characteristics illustrate that the current transformer‐based neural architecture with specialty‐based fine‐tuning with curation from peer‐reviewed resources for a specialty (in this case, gastroenterology) can lead to the development of a precision AI model with clinical relevance.

**TABLE 1:** A comparison matrix between GastroGPT and other LLM models.

Clinical task	GastroGPT (mean ± SD)	Best general model (mean ± SD; model)	Absolute difference	Highest score (yes/no)	Interpretation	Task complexity/importance in gastroenterology
Assessment and summary	7.91 ± 1.70	7.89 ± 1.71 (Claude)	+0.02	Yes	Comparable performance, high reliability	Fundamental for diagnosis; guides initial management
Additional history gathering	8.43 ± 1.83	2.98 ± 3.05 (GPT‐4)	+5.45	Yes	Marked superiority, much greater consistency	High complexity; essential for uncovering risk and narrowing diagnosis
Recommended diagnostic studies	7.90 ± 1.77	7.36 ± 1.82 (Claude)	+0.54	Yes	Moderate advantage, reliable output	Key for evidence‐based investigation and cost‐effective care
Proposed management plan	7.97 ± 2.09	7.73 ± 2.11 (Bard)	+0.24	Yes	Slightly better and consistent	Critically important; requires adaptation to evolving guidelines
Follow‐up planning	7.51 ± 2.00	7.84 ± 1.98 (Claude)	−0.33	No	Slightly lower than best general model	Ensures continuity of care, crucial for chronic conditions
Referral guidance	8.30 ± 1.67	7.77 ± 1.66 (Claude)	+0.53	Yes	Superior accuracy and better consistency	High complexity; enables multidisciplinary, comprehensive care
Patient counseling/communication	8.50 ± 1.73	7.87 ± 1.81 (Claude)	+0.63	Yes	Clear advantage with consistent performance	Central for shared decision‐making and patient outcomes
Overall quality of assessment	8.34 ± 1.29	7.89 ± 1.40 (Claude)	+0.45	Yes	Overall expert alignment favoring GastroGPT	Integrates & reflects total expert confidence in model guidance

## 3 Limitations and Challenges: Tempering the AI Enthusiasm

The use of 10 simulated cases in the study restricts generalizability, as the variability of real‐world patients, including comorbidities and differing interactions, has not been examined yet. It remains unclear whether GastroGPT is enhancing expert performance or simply approximating it in the absence of direct comparisons with human clinicians, and this gap in evaluations of LLMs is extremely crucial. During the compilation of the training corpus, common conditions may dominate and lead to inaccuracies or hallucinations, due to the underrepresentation of rare conditions, as has been identified in wider AI research [7]. According to a recent study identifying deskilling risks of reliance on AI, continuous exposure in colonoscopy was associated with lower adenoma detection rates in non‐AI cases [8]. GastroGPT's similar performance with Bard in follow‐up planning (*p* = 0.16) and slightly better performance in diagnostic studies of primary care suggest there is room for enhancements, one of which may involve integration of multimodal data [6]. While the model will depend on a trained dataset, there will be another challenge in the form of clinical outdating of standards, if the model is not periodically trained and validated. Ethical issues, including privacy of data and equitable deployment, should also be considered to avoid developing further inequity in healthcare. It is significant that the study did not detail comparisons to OpenEvidence, a medical‐specific LLM, missing an opportunity to compare GastroGPT with a clinically focused model and also underlining the importance of wider evaluations to validate a specific AI in gastroenterology.

## 4 Broader Implications and Future Direction: From Proof of Concept to Practice

**FIGURE 1:**
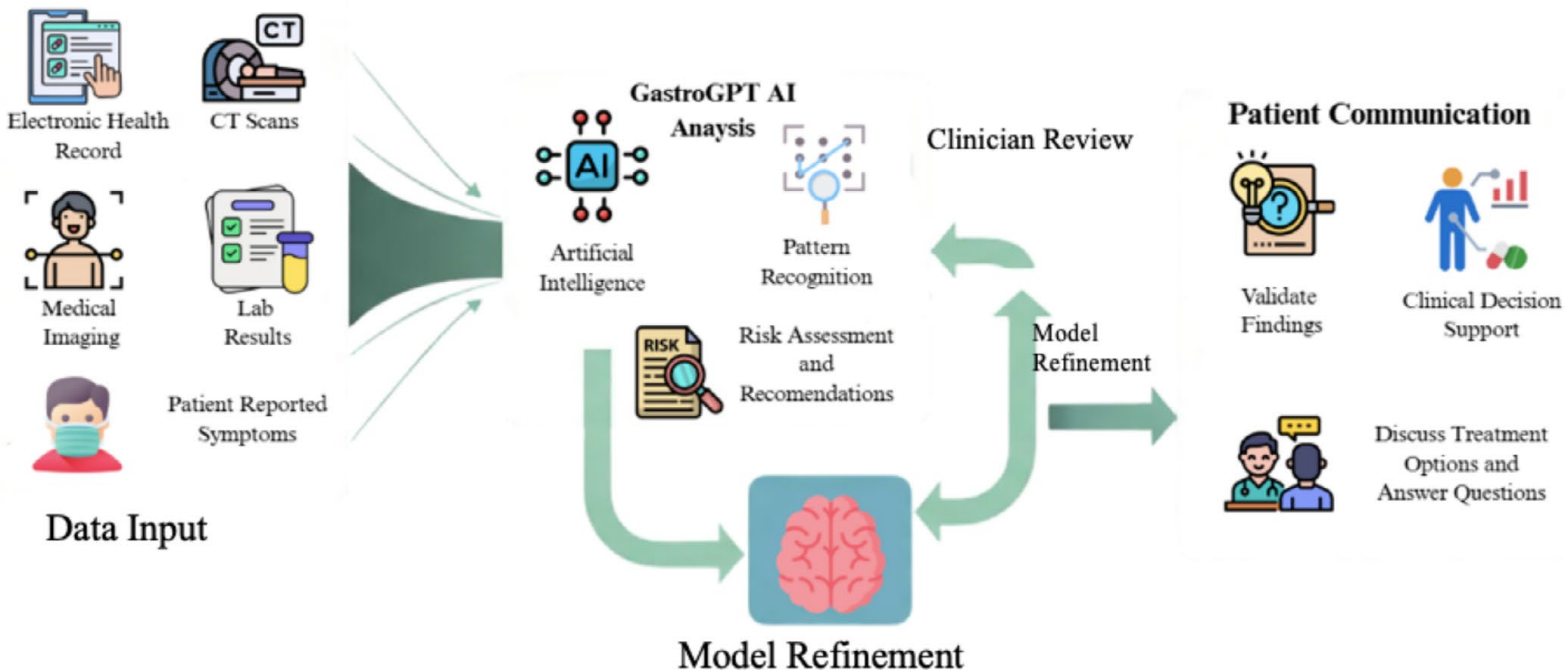
Clinical integration workflow.

Targeted models such as GastroGPT could revolutionize clinical practice workflows by facilitating triage, multidisciplinary coordination and patient‐centered communication within gastroenterology [9, 10]. A clinical integration workflow has been depicted in Figure 1. In fields like hepatology and precision endoscopy, AI will improve guideline‐concordant management of conditions such as chronic pancreatitis or hepatocellular carcinoma, especially in underserved areas. This aligns with trends toward augmented intelligence; AI can complement human intelligence in order to mitigate provider shortages and improve diagnostic accuracy. Regulatory systems with hybrid model types, such as medical devices that use AI, are necessary to weigh innovation against safety. Hybrid models that involve specialized and general LLMs could optimize their performance by taking advantage of an LLM's capacity for domain‐specific accuracy with an LLM's capacity for broad reasoning capabilities. This could be employed for specific specialties such as radiology or oncology or could lead to even a broader impact of AI in healthcare delivery in general.

Multicentre prospective real‐world trials that engage patient populations from diverse communities and multimodal data sources, such as endoscopic imaging, will be needed to confirm GastroGPT's benefits. Updating the AI model with the most current clinical practice guidelines will help minimize obsolescence, while using hybrid architectures has the potential to overcome limitations on task‐specific generative outputs. Hybridizing GastroGPT with clinical computational solutions like BENEIN, which identifies master regulators, such as MYB, HDAC2 and FOXA2 to reverse colorectal cancer cells to normal enterocytes could provide LLMs with molecular insights that inform innovative clinical decisions in differentiating therapies or patient counseling regarding treatment plans [11]. Standards and metrics to assess indications of bias, safety and clinical‐related outcomes are central to ethical implementation. Models that promote cross‐specialties or subspecialties also have the potential to establish the framework for the development of specialty‐derived AI technologies for use in medicine.

## 5 Conclusion

GastroGPT defines a major step in the development of domain‐specific AI designed for gastroenterology, showing superior performance and reliability compared to generalist LLMs in simulated scenarios. Real‐world validation, multimodal integration, and comparisons with newer medical domains will be important to pursue in future research that can delineate the transformative role of domain‐specific models in healthcare delivery.

## Conflicts of Interest

The authors declare no conflicts of interest.

## Data Availability

Data sharing not applicable to this article as no datasets were generated or analyzed during the current study.
